# Emotional intelligence and reflective thinking: a synergistic approach in nursing education

**DOI:** 10.1186/s12912-025-03175-w

**Published:** 2025-05-16

**Authors:** Rawhia Salah Dogham, Nermine M. Elcokany, Asmaa Saber Ghaly, Ayman Mohamed El-Ashry, Heba Fakieh Mansy Ali

**Affiliations:** 1 Nursing Education Department, Faculty of Nursing, Alexandria University, Alexandria, Egypt; 2 Critical Care & Emergency Nursing, Faculty of Nursing, Alexandria University, Alexandria, Egypt; 3 Obstetrics and Gynecology Nursing Department, Faculty of Nursing, Alexandria University, Alexandria, Egypt; 4 Psychiatric and Mental Health Nursing Department, Faculty of Nursing, Alexandria University, Alexandria, Egypt

**Keywords:** Emotional intelligence, Nursing education, Reflective thinking, Nursing students

## Abstract

**Background:**

Emotional intelligence (EI) is crucial in stress management and overall well-being. Emotions are integral to delivering high-quality clinical care, as they facilitate reflective thinking, which is essential for effective nursing practice.

**Objective:**

This study assessed the relationship between emotional intelligence and reflective thinking among nursing students.

**Design:**

A cross-sectional correlational design was employed.

**Methods:**

A stratified random sample of 1,096 nursing students participated in the study. Data were collected using the Trait Emotional Intelligence Questionnaire-Short Form and the Reflective Thinking Questionnaire.

**Results:**

The findings revealed that 57.0% of nursing students demonstrated high levels of emotional intelligence, with the well-being domain scoring the highest. Additionally, 59.3% exhibited high levels of reflective thinking, particularly in understanding and reflection. A significant positive correlation was identified between emotional intelligence and reflective thinking (*r* = 0.612, *p* < 0.001). All domains of emotional intelligence—emotionality, self-control, sociability, and well-being—were strongly associated with reflective thinking.

**Conclusion:**

The study concludes that emotional intelligence and reflective thinking are positively correlated among nursing students, with higher emotional intelligence levels linked to enhanced reflective thinking skills. These findings highlight the importance of integrating emotional intelligence and reflective thinking training into nursing education to strengthen emotional and cognitive competencies and better prepare students for clinical practice.

**Implications for nursing and health policy:**

Nursing curricula should incorporate formal emotional intelligence training as a foundational education component. Given the positive association between EI, reflective thinking, and decision-making, EI modules can help students develop critical emotional competencies, such as self-regulation, empathy, and interpersonal skills. Educational institutions should guide students in cultivating these skills to enhance their reflective thinking and critical decision-making abilities during clinical practice. This integration will ultimately improve patient care outcomes and foster professional resilience among nursing students.

**Clinical trial number:**

Not applicable.

## Introduction

Nursing, a profession that involves significant emotional labor, necessitates direct, daily interaction with individuals. The preparation of nursing students to become competent professional nurses is a key priority for nursing colleges. The holistic and compassionate care provided by nurses stems not only from their knowledge of disease conditions but also from their emotional intelligence. In today’s ‘age of uncertainty,’ nurses must possess strong socio-emotional competencies to navigate complex and emotionally demanding environments. This underscores the crucial role of emotional intelligence in nursing [1, 2].

Emotional intelligence (EI) is a crucial construct in nursing education [3]. It is the ability to monitor one’s own and others’ emotions, discern between positive and negative consequences of emotions, and use emotion-related information to drive cognitive reasoning and actions. EI acts as a bridge between emotions and logical thought, linking critical interpersonal and personal skills. The five qualities that make up EI-self-awareness, managing emotions, motivating oneself, empathy, and social skills-are essential for nursing students to navigate the emotional demands of the profession [3].

There are four dimensions of emotional intelligence - well-being, self-control, emotionality, and sociability. Emotional facets include empathy, emotional expression, emotional perception, and relationships. Self-control, as a dimension of emotional intelligence, consists of low impulsiveness, stress, management, and emotion regulation. Sociability as a dimension of emotional intelligence includes assertiveness, emotion management, communication, social awareness, self-esteem, and communication. Well-being is a dimension of emotional intelligence measured by three aspects: trait happiness, self-esteem, and trait optimism [4].

EI has been especially significant in nursing in recent years because of its beneficial effects on patients, healthcare providers, and organizations. Research shows that EI enhances communication and builds inter-professional partnerships, which reduce stress and avoid nurse burnout [5, 6]. These advantages improve patient care safety and quality by influencing the larger practice environment and individual performance. Additionally, EI growth is linked to higher job satisfaction and retention rates among healthcare professionals, underscoring its vital role in fostering general well-being and efficacy in healthcare settings. This potential of EI to significantly improve patient care and healthcare professionals’ well-being is a reason for optimism in the nursing field [7, 8].

Additionally, managing and expressing pertinent emotions is a significant emotional task in nursing. To advance the nursing profession, researchers and educators must concentrate on enabling EI in nurses. High EI nurses are more resilient, sympathetic, caring, and empathetic [9]. EI might be significant in nursing students’ retention and achievement [10]. It can help nursing students efficiently handle difficulties in clinical placements, boost patient safety, and improve their leadership and practice performance [11].

Furthermore, it was discovered that nursing students with high EI performed well academically and experienced less academic stress [12]. To provide safe and efficient patient care, nurses must handle emotionally charged circumstances requiring reflective thinking skills [13]. Because reflective thinking abilities are closely linked to reducing unfavorable patient outcomes, nursing education prioritizes their development [14].

Reflective thinking is a critical aspect of nursing education, involving the analysis of one’s thoughts, experiences, and emotions to gain deeper insights and understanding. It encourages self-evaluation and promotes a growth mindset, which is vital for personal and professional development [15]. Reflective thinking is a manageable process that can be strengthened and developed using various instructional strategies in nursing education, such as case discussions, clinical experiences, reflective writing, Socratic questioning, simulation, and games [16].

Reflection has four levels: non-reflection, understanding, reflection, and critical reflection. Non-reflection occurs when a learner presents material without attempting to understand or interpret it, often reproducing others’ work without deeper engagement. Understanding indicates comprehension of a concept but remains limited to theoretical knowledge, relying on textbooks or lecture notes without connecting to real-life applications. Reflection moves beyond theory by applying it to practical situations, allowing for personal insights beyond book learning. Critical reflection is the most advanced level, involving a fundamental shift in perspective regarding a key concept, though such deep transformations are rare [17]. Reflective thinking is a metacognitive process that occurs before, during, and after a situation to better comprehend the person and the circumstance in which they find themselves.

Furthermore, it makes a person more aware of their performance, strengths, and weaknesses in that specific circumstance. As a result, individuals will be exposed to more situations that promote learning and personal growth, ultimately enhancing their skills and performance [6]. Additionally, reflective thinking helps learners track their progress from novice to expert and gives nurses a practical means of analyzing and assessing their learning processes [11].

Enhancing reflective thinking and clinical judgment in nursing students is essential for increasing patient care quality, lowering stress levels associated with nursing, and boosting students’ mental well-being [18]. EI directs thought processes and reflective thinking, enabling logical choices and efficient problem-solving that benefit patients and healthcare professionals. Strong EI abilities are necessary to navigate emotionally charged situations since emotions greatly influence judgment, decision-making, and reflective thinking. Effective clinical nursing intervention requires striking a balance between emotions and rationality [19].

While the relationship between emotional intelligence and other aspects has been studied, there is still much to learn about how emotional intelligence and reflective thinking are related, particularly among Egyptian undergraduate nursing students. Research has shown that nursing students’ clinical decision-making, critical thinking, academic stress management, and problem-solving abilities positively correlate with emotional intelligence. Given the interconnected nature of emotional intelligence and reflective thinking, it is crucial to understand how these concepts relate to one another in the context of nursing students. This understanding can enhance nursing students’ performance and is the focus of the current study.

By addressing this research gap, the study aims to offer valuable insights into the relationship between emotional intelligence (EI) and reflective thinking among Egyptian undergraduate nursing students. The findings could help future nurses better prepare for the profession’s demands, improve academic performance, and reduce stress, ultimately enhancing their readiness for clinical practice.

### Research objective

- Identify the relationship between emotional intelligence and reflective thinking among nursing students.

### Research design

A cross-sectional correlational research design following Strengthening the Reporting of Observational Studies in Epidemiology (STROBE) guidelines was utilized for this study.

### Setting

The study was conducted at Alexandria University’s College of Nursing. The college follows a credit-hour system for its undergraduate and graduate programs. Undergraduate students pursuing a baccalaureate degree in nursing sciences are required to complete a four-year, comprehensive program divided into eight semesters.

### Participants and sample size calculation

To determine the appropriate sample size, the researchers utilized the Epi Info 7 software, inputting specific parameters including a total population of 5,520 students for the 2024–2025 academic year, an anticipated frequency of 50%, a margin of error set at 5%, and a 95% confidence level. Based on these criteria, the program recommended a minimum sample size of 359 students. However, to enhance the study’s accuracy and reliability, the researchers opted for a larger sample size of 1,096 nursing students, employing a stratified random sampling method. The students were categorized into four strata according to their academic year: 316 from the first year, 308 from the second year, 272 from the third year, and 200 from the fourth year. This expanded sample size contributed to greater precision and strengthened the overall robustness of the study.

### Study tools

#### Tool I: Trait Emotional Intelligence Questionnaire-Short Form (TEIQue-SF)

TEIQue-SF was developed by Petrides (2009). The researchers used it to assess nursing students’ emotional intelligence. The questionnaire is a 30-item self-report test that assesses global trait emotional intelligence. The questionnaire includes 15 reversed items, which are 16, 2, 18, 4, 5, 7, 22, 8, 10, 25, 26, 12, 13, 28, and 14. The questionnaire is divided into four domains: emotionality, which consists of eight statements; self-control, which consists of ten statements; Sociability, which consists of six statements; and well-being, which consists of six statements. Students use a 7-point Likert scale to score the frequency with which they encounter a particular situation on each of the 30 items. The scale ranges from “completely disagree” (1 point) to “completely agree” (7 points). The global trait emotional intelligence score is computed using the mean of all 30 responses. The TEIQue-SF data were analyzed using the following scoring system: Above-average percent scores range from 70 to 99%; average percent scores are 30-69%; and below-average percent scores are 1-29%. The internal consistency of the TEIQue-SF was discovered to be strong, with alpha coefficients ranging from 0.87 to 0.89 [20].

#### Tool II: Reflective Thinking Questionnaire (RTQ)

This questionnaire was designed by Kember et al. (2000) to assess nursing students’ reflective thinking. The questionnaire consists of 16 items rated on a 5-point Likert scale, ranging from 5 “Definitely Agree” to 1 “Definitely Disagree,” with intermediate options such as 4 “Agree with reservation,” 3 “Neutral,” and 2 “Disagree with reservation.” The questionnaire has four subscales: habitual action, understanding, reflection, and critical reflection. Each subscale’s minimum and maximum scores are four and twenty, respectively. Kember et al. (2000) discovered that the Cronbach’s alpha for the four subscales ranged between 0.62 and 0.76, indicating acceptable internal consistency [21].

In addition, personal and academic characteristics include students’ age, gender, marital status, academic level, work during education, previous qualifications, and latest attained Grade Point Average (GPA).

### Ethical considerations

The Research Ethical Committee (REC) of the College of Nursing at Alexandria University (**IRB00013620-2024**) approved the study before it began. By ethical rules, all potential participants were entirely told about the study’s goal, voluntary nature, and the possibility of withdrawing at any moment with no consequences. All participants provided informed written consent, ensuring they were fully aware of the study’s objectives and procedures and their right to withdraw at any time without penalty while maintaining the confidentiality and anonymity of their responses.

To maintain confidentiality and anonymity, the researchers employed several measures throughout the data collection and analysis processes. No personal identifying information (such as names or contact details) was collected from participants. Instead, each participant was assigned a unique code to ensure that responses remained anonymous. The completed questionnaires were kept in a secure, locked environment and were accessible only to the research team. The data were stored in password-protected files and were treated with the utmost confidentiality.

### Validity and reliability

To ensure the accuracy, cultural appropriateness, and structural validity of the tools used in this study, a rigorous process of translation, back-translation, and the Lewen test was conducted. The original English versions of the Trait Emotional Intelligence Questionnaire-Short Form (TEIQue-SF) and the Reflective Thinking Questionnaire (RTQ) were translated into Arabic by a bilingual expert fluent in English and Arabic. The translated Arabic versions were then independently back-translated into English by a second bilingual expert without prior knowledge of the original tools. The back-translated English versions were compared with the original instruments to identify and resolve any discrepancies in meaning, ensuring that the Arabic translations retained the intended concepts and nuances of the original tools.

To further validate the translation process, a Lewen test (Loevinger’s H coefficient) was conducted to assess the scalability and homogeneity of the translated items. The Lewen test produced a scalability coefficient (H) for each tool, with values of 0.42 for the TEIQue-SF and 0.38 for the RTQ. These values, both exceeding the acceptable threshold of 0.30, confirm that the items in the translated Arabic versions are well-structured, scalable, and homogeneous, demonstrating strong structural validity. Additionally, Cronbach’s alpha was calculated to evaluate internal consistency, yielding values of 0.87 to 0.89 for the TEIQue-SF and 0.62 to 0.76 for the RTQ, further confirming the reliability of the tools. These results collectively ensure that the translated Arabic versions of the tools are valid, reliable, and culturally appropriate for measuring emotional intelligence and reflective thinking among Arabic-speaking nursing students.

### Pilot study

Following a meticulous validation process, a pilot study was executed with 10% of the nursing student population (*n* = 110) to evaluate the instruments’ feasibility, clarity, and applicability. The pilot study outcomes revealed that participants found the tools to be clear, relevant, and appropriate for the study’s context. The constructive feedback gathered was instrumental in refining the instruments, ensuring they were both user-friendly and straightforward to administer. To maintain the integrity of the primary study, participants from the pilot study were deliberately excluded to prevent bias. This thorough and methodical approach—which included translation, back-translation, the Lewen test, Cronbach’s alpha analysis, and a pilot study—guaranteed the tools’ accuracy, reliability, and cultural relevance, ultimately making them exceptionally suited for the main study.

### Data collection

Before initiating data collection, the researchers organized face-to-face group interviews with the recruited students in their classrooms, either after lectures or during clinical sessions. The researchers introduced themselves and explained the study’s purpose, objectives, and significance. To protect students from any perceived obligation or coercion—particularly because some members of the research team are also faculty at the College of Nursing—special measures were implemented. In cases where a researcher had a direct teaching role with a student group, data collection was either conducted by other non-teaching faculty or scheduled at times when the instructor was not involved. This helped ensure a neutral and pressure-free environment. Additionally, all responses were anonymous, and no identifying information was collected, further protecting participant confidentiality.

For data collection, the researchers distributed printed questionnaires to the nursing students during these face-to-face sessions. They provided clear instructions on completing the questionnaire and remained present to address questions, clarify doubts, or offer additional explanations. This approach ensured that students fully understood the survey items and could provide accurate and meaningful responses. The researchers also collected the completed questionnaires immediately after completion to maintain data integrity and prevent loss or incomplete submissions. This face-to-face method fostered a supportive environment, encouraged higher participation rates, and ensured the reliability and completeness of the collected data.

### Data analysis

This study employed IBM SPSS version 20.0 for comprehensive statistical analysis. Categorical data were effectively represented as frequencies and percentages, while continuous data were rigorously tested for normality using the Shapiro-Wilk test. The results were clearly summarized, showcasing the range, mean, standard deviation, median, and interquartile range. A significance level of 5% was established to ensure robust findings. In the comparison of normally distributed quantitative variables between two groups, the Student’s t-test was utilized. The F-test (ANOVA) was conducted for comparisons among multiple groups, complemented by a post hoc Tukey test for insightful pairwise comparisons. Pearson’s correlation coefficient was employed to uncover relationships between two quantitative variables, and a linear regression analysis was performed to elucidate the influence of emotional intelligence on reflective thinking while controlling for personal and academic characteristics. This comprehensive approach ensures the reliability and validity of our findings, making a compelling case for the importance of emotional intelligence in reflective thinking.

## Results

Table 1 shows that the majority (62.7%) were female, and about two-thirds (65.7%) were aged 20 or older, with a mean age of 20.15 ± 1.67 years. In terms of academic level, the largest group (28.9%) were first-year students, followed by second-year (28.1%), third-year (24.8%), and fourth-year (18.2%) students. Most students were single (89.4%), and about two-thirds (60.4%) were not working while studying. Regarding academic performance, more than half (55.5%) had a B- to B + GPA, while only 17.0% achieved an A- to A. Additionally, 60.7% of students held a secondary school certificate as their previous qualification.

**Table 1:** Distribution of the nursing students according to their personal and academic characteristics

Personal and academic characteristics	Nursing students (*n* = 1096)	
	No.	%
**Academic year**		
First	316	28.9
Second	308	28.1
Third	272	24.8
Fourth	200	18.2
**Gender**		
Male	409	37.3
Female	687	62.7
**Age**		
< 20	376	34.3
≥ 20	720	65.7
Mean ± SD.	20.15 ± 1.67	
**Marital status**		
Single	999	91.1
Married	94	8.6
Divorced	3	0.3
**Do you work during education?**		
No	662	60.4
Yes	434	39.6
**Last obtained GPA**		
A- – A	186	17.0
B- – B+	608	55.5
C- – C+	302	27.6
D – D+	0	0.0
F	0	0.0
Mean ± SD.	3.0 ± 0.61	
**Previous qualification**		
Technical school certificate	236	21.5
Secondary school certificate	665	60.7
Other faculty	195	17.8

SD: Standard deviation

Table 2 displays the students’ scores across the four domains of emotional intelligence. The emotionality domain scores ranged from 11.0 to 55.0, with a mean of 38.73 ± 6.99 and a mean percent score of 64.01 ± 14.57. The self-control domain had a mean score of 48.42 ± 8.76 (mean percent score: 64.03 ± 14.59), while sociability had a mean score of 29.74 ± 5.57 (mean percent score: 65.94 ± 15.48). The well-being domain had a mean score of 30.79 ± 6.01 (mean percent score: 68.87 ± 16.68). Regarding the four subscales of reflective thinking; the habitual action subscale scores ranged from 4.0 to 20.0, with a mean of 13.06 ± 3.19 (mean percent score: 56.63 ± 19.95). The understanding subscale had scores ranging from 4.0 to 20.0, with a mean of 16.82 ± 2.37 (mean percent score: 80.13 ± 14.79). The reflection subscale scores ranged from 6.0 to 20.0, with a mean of 16.43 ± 2.40 (mean percent score: 77.70 ± 15.02). Finally, the critical reflection subscale scores ranged from 7.0 to 20.0, with a mean of 15.49 ± 2.69 (mean percent score: 71.80 ± 16.79).

**Table 2:** Nursing students’ scores on the domains of emotional intelligence and subscales of reflective thinking

	Total score	Percent Score
	Mean ± SD.	Mean ± SD.
Emotionality	38.73 ± 6.99	64.01 ± 14.57
Self-Control	48.42 ± 8.76	64.03 ± 14.59
Sociability	29.74 ± 5.57	65.94 ± 15.48
Well being	30.79 ± 6.01	68.87 ± 16.68
Overall	147.68 ± 23.24	65.38 ± 12.91
**Reflective thinking**		
Habitual Action	13.06 ± 3.19	56.63 ± 19.95
Understanding	16.82 ± 2.37	80.13 ± 14.79
Reflection	16.43 ± 2.40	77.70 ± 15.02
Critical reflection	15.49 ± 2.69	71.80 ± 16.79
Overall	61.80 ± 7.42	71.56–11.60

SD: Standard deviation

Domains of Emotional Intelligence Mean Percent score interpretations

Above Average: 70- 99%, Average: 30-69%, 1-29%: Below Average

**Fig. 1:**
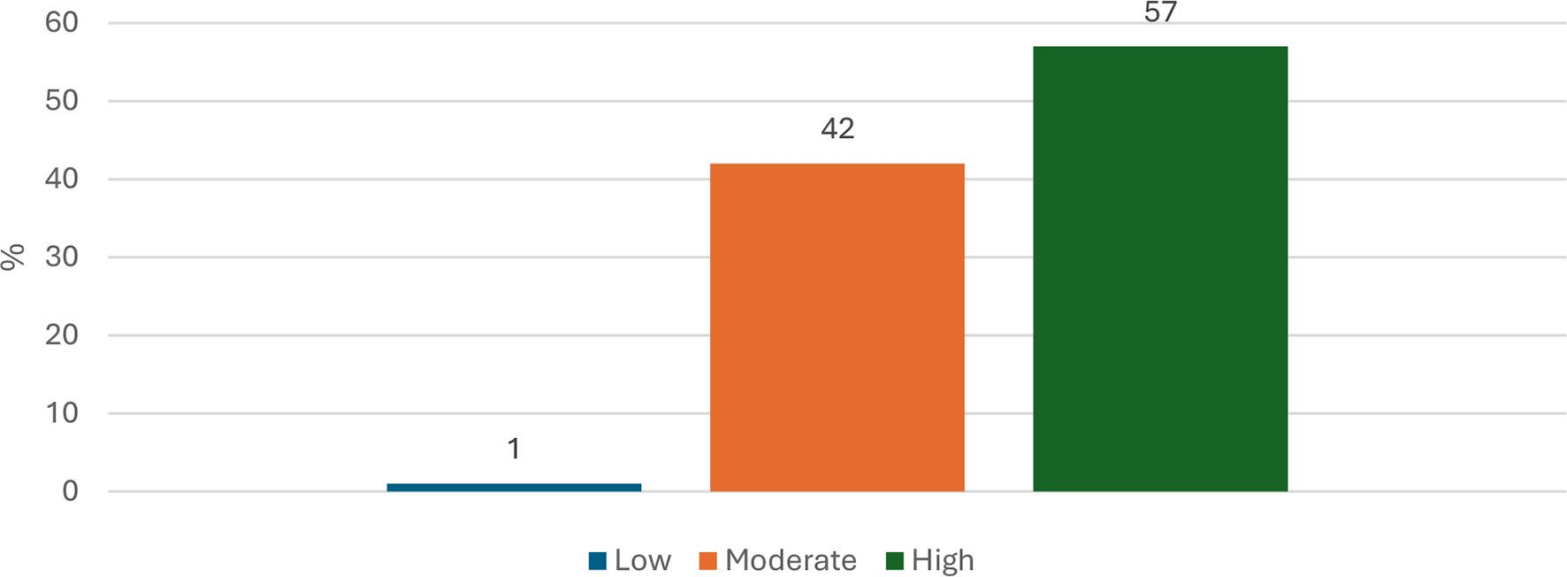
Frequency distribution of the nursing students according to their level of emotional intelligence

Figure 1 illustrates the students’ emotional intelligence levels. Nearly all students fell into either the high (57.0%) or moderate (42.0%) emotional intelligence categories.

**Fig. 2:**
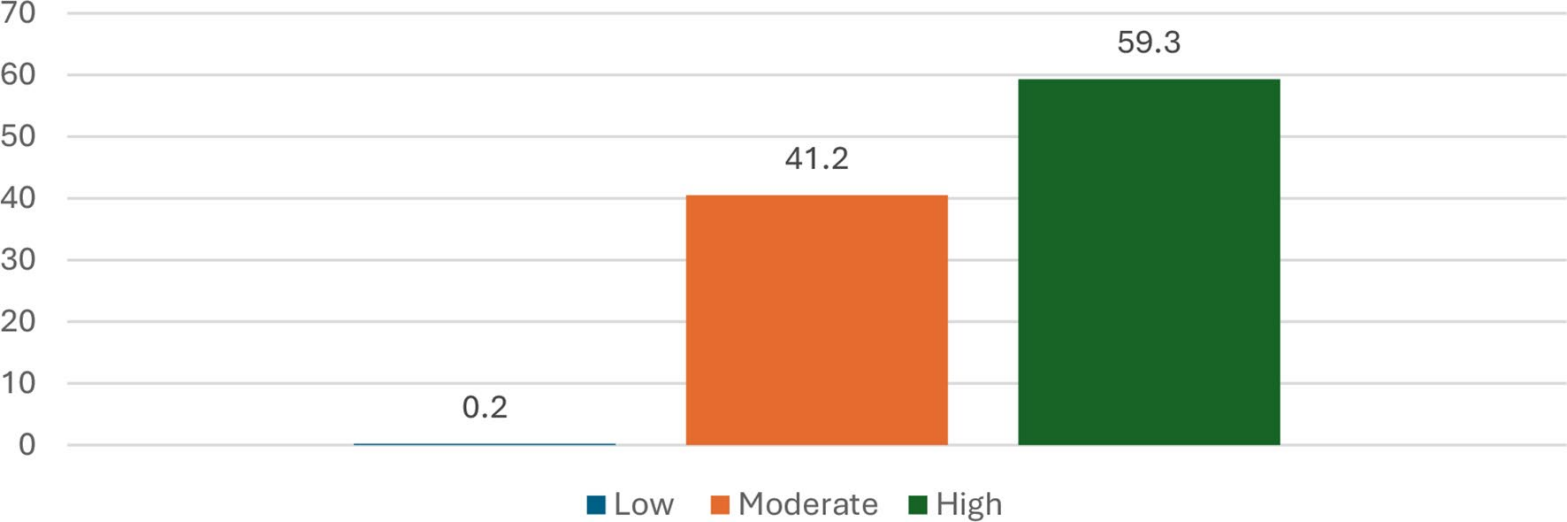
Distribution of the nursing students according to their levels and scores of reflective thinking

Figure 2 shows the students’ reflective thinking levels. 59.3% of students had a high level of reflective thinking, while only 0.2% had a low level.

**Fig. 3:**
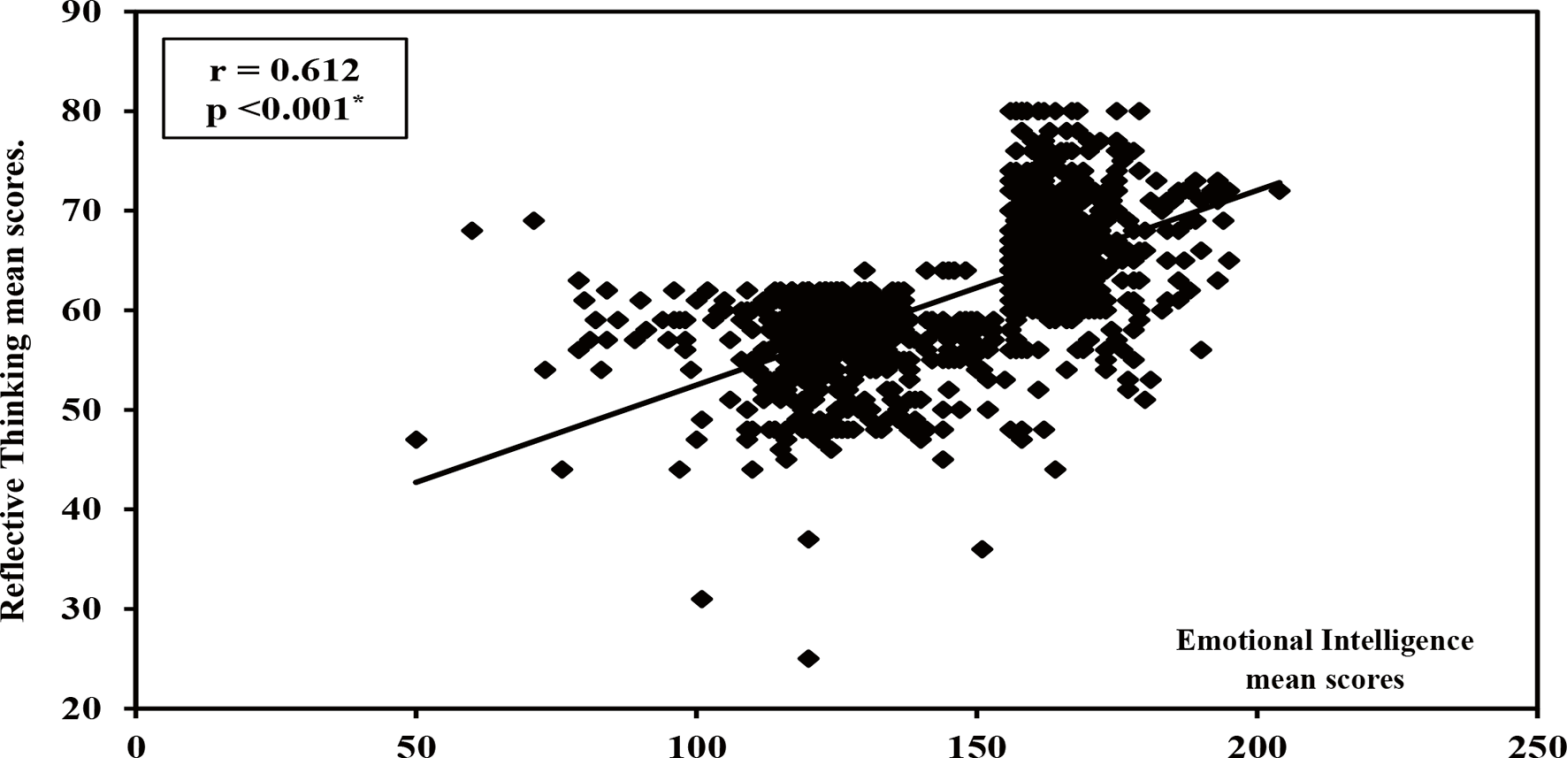
Correlation between mean scores of the nursing students’ emotional intelligence and their reflective thinking

Figure 3 highlights the correlation between students’ emotional intelligence and reflective thinking. A statistically significant positive correlation was found between overall emotional intelligence and reflective thinking (*r* = 0.612, *p* < 0.001*). Additionally, all four emotional intelligence subscales were positively and significantly correlated with the four subscales of reflective thinking (*p* < 0.001* for each subscale).

Table 3 examines the correlation between students’ personal and academic characteristics and their reflective thinking and emotional intelligence. A statistically significant correlation was found only between students’ previous qualifications and emotional intelligence (*p* = 0.020*). No significant correlations were observed between gender, age, academic level, marital status, employment during education, or GPA and reflective thinking or emotional intelligence (*p* = 0.238, 0.196, 0.457, 0.232, 0.136, 0.488, 0.538, 0.194, 0.072, 0.349, and 0.472, respectively). Furthermore, no significant correlation was found between previous qualifications and reflective thinking (*p* = 0.573).

**Table 3:** Correlation between nursing students’ personal and academic characteristics and their reflective thinking and emotional intelligence

Personal and academic characteristics	Reflective thinking	Emotional intelligence
	Mean ± SD.	Mean ± SD.
**Gender**		
Male	61.46 ± 7.69	146.50 ± 23.49
Female	62.00 ± 7.26	148.39 ± 23.08
**t (p)**	**1.181 (0.238)**	**1.294 (0.196)**
**Age**		
< 20	61.57 ± 7.40	146.52 ± 23.72
≥20	61.92 ± 7.44	148.28 ± 22.98
**t (p)**	**0.745 (0.457)**	**1.196 (0.232)**
**Academic year**		
First	61.55 ± 7.05	146.15 ± 23.73
Second	61.45 ± 8.36	147.57 ± 22.82
Third	62.72 ± 6.57	148.88 ± 22.52
Fourth	61.49 ± 7.50	148.63 ± 24.08
**F (p)**	**1.852 (0.136)**	**0.810 (0.488)**
**Marital status**		
Single	61.70 ± 7.24	147.51 ± 23.31
Married	62.74 ± 8.76	150.05 ± 21.34
Divorced	65.67 ± 6.51	157.00 ± 39.34
**F (p)**	**0.844 (0.470)**	**0.724 (0.538)**
**Do you work while studying?**		
No	61.57 ± 7.55	146.68 ± 24.07
Yes	62.16 ± 7.21	149.21 ± 21.85
**t (p)**	**1.301 (0.194)**	**1.799 (0.072)**
**Last obtained GPA**		
A- – A	61.66 ± 7.04	147.11 ± 23.15
B- – B+	62.08 ± 7.45	148.43 ± 22.70
C- – C+	61.33 ± 7.59	146.52 ± 24.36
D – D+	0	0
F	0	0
**F (p)**	**1.055 (0.349)**	**0.751 (0.472)**
**Previous qualifications**		
Technical nursing school certificate	61.66 ± 7.42	150.52 ± 22.74
Secondary general school certificate	61.70 ± 7.37	146.12 ± 23.65
Others faculty	62.31 ± 7.63	149.55 ± 22.06
**F (p)**	**0.557 (0.573)**	**3.912** ^*^ **(0.020** ^*^ **)**

SD: Standard deviation t: Student t-test F: F for One way ANOVA test

p: p value for comparison between the studied categories

*: Statistically significant at *p* ≤ 0.05

Table 4 explores potential explanatory factors for emotional intelligence and reflective thinking. A multivariate linear regression analysis revealed that emotional factors (emotionality, self-control, well-being, and sociability) had significant associations (*p* < 0.05) with reflective thinking, while demographic and academic variables did not. Emotionality and self-control showed the most substantial relationships (B = 0.244), indicating that these psychological factors are more influential than demographic or academic variables.

**Table 4:** Multivariate linear regression analysis of predictive factors regarding emotional intelligence with reflective thinking

	Multivariate	
	Reflective thinking	
	*p*	B (LL – UL 95%C.I)
**Gender**	0.457	-0.131 (-0.476–0.214)
**Age**	0.293	0.425 (-0.367–1.217)
**Academic year**	0.782	0.032 (-0.194–0.258)
**Marital status**	0.508	0.214 (-0.421–0.849)
**Do you work during education?**	0.721	0.139 (-0.625–0.904)
**GPA**	0.536	0.184 (-0.401–0.770)
**Previous qualifications**	0.153	0.443 (-0.166–1.052)
**Emotional intelligence**		
Emotionality	< 0.001^*^	0.244 (0.175–0.313)
Self-Control	< 0.001^*^	0.244 (0.180–0.307)
Sociability	0.041^*^	0.096 (0.004–0.188)
Well-being	< 0.001^*^	0.153 (0.070–0.236)

Durbin-Watson = 2.012; VIF= (1.065–2.584); F = 60.856; *P* < 0.001^*^R = 0.618; R^2^ = 0.382; AdjR^2^ = 0.376

B: Unstandardized Coefficients C.I: Confidence interval LL: Lower limit UL: Upper Limit

*: Statistically significant at *p* ≤ 0.05

## Discussion

This cross-sectional correlational study explored the EI and reflective thinking levels among nursing students. Nursing students have high levels of both EI and reflective thinking. The findings revealed a strong positive correlation between the overall emotional and reflective thinking scores of nursing students. Moreover, reflective thinking is positively influenced by emotionality, self-control, sociability, and well-being.

The findings indicated that more than half of the students had high levels of EI, with well-being scoring the highest among the domains. This aligns with Shanta, & Gargiulo, (2014), who emphasized that nursing students frequently display high levels of EI due to the empathetic and interpersonal demands of their education and training [22]. MacCann et al., (2020) reported that nursing students demonstrate relatively high levels of emotional intelligence, which aids stress management and enhances their academic and clinical performance [23]. In addition, Dugué et al., (2021) reported in their study that nursing students exhibit high EI levels due to the integration of EI-related content into nursing education programs.

The current study findings reveal that almost half of nursing students’ EI falls between moderate and low EI scores, and this might be due to factors such as stressors in the academic environment that nursing students encounter during their journey suggest an opportunity for targeted improvement, particularly in domains like sociability and self-control. These findings are in line with Rodríguez-Leal et al., (2023) study, which reported that a significant proportion of nursing students scored low to moderate in emotional intelligence, which correlated with high levels of clinical stress.

The study findings show that reflective thinking scores were highest in understanding and reflection domains, indicating students’ firm conceptual grasp and evaluative capabilities. In line with the current study, Smith, (2021) highlighted that nursing students often demonstrate high levels of reflective thinking, mainly when guided by structured reflective strategies in their education [24]. Moreover, Bjerkvik, Hilli, (2019) study highlighted that reflective journaling might enhance reflective thinking in nursing students, particularly in evaluating their experiences and improving clinical practice [25]. In addition, reflective thinking was underdeveloped in nursing students, with many relying on structured tools or guidance rather than independent critical reflection [26].

Other researchers have studied the relationship between EI and psychological status or critical thinking among nursing students. However, the relationship between EI and reflective thinking is unknown. The current study findings highlighted a positive correlation between students’ overall EI and reflective thinking. Moreover, the four subscales of EI are positively and significantly correlated with the four subscales of reflective thinking. The interplay between reflective thinking and EI creates a synergistic relationship that promotes emotional and intellectual growth. This might be due to the interconnected nature of these competencies in fostering self-awareness, critical analysis, and adaptive learning.

Reflective thinking involves cognitive processes such as critical reasoning, evaluation, and synthesis. Emotional intelligence (EI) complements these processes by providing the emotional stability and awareness needed for meaningful reflection. Reflective thinking arises from the interplay between critical and creative thinking within the framework of integrated thought. This interplay suggests that vital, reflective, and innovative thinking are positively and significantly interrelated [15]. Studies by Hasan and Noor (2024) reported a strong positive correlation between overall EI and critical thinking disposition. Similarly, Zuhal, (2012) found a significant relationship between EI and critical thinking. Critical, reflective, and creative thinking form a synergistic relationship fostering emotional and intellectual growth [27].

Emotional intelligence, particularly in emotionality and self-control, enables students to recognize, regulate, and reflect on their emotions. These findings align with Bsharat, (2024) study, which identified a moderately positive relationship between EI and the ability to utilize emotions among fourth-year nursing students. This highlights the importance of EI in enhancing reflective thinking and overall cognitive development.

The lack of significant correlations between gender, age, or academic level with EI and reflective thinking suggests these competencies are not inherently tied to demographic factors. These findings contradict those of Budlar et al. (2022); who found that EI tends to increase with age and progression through academic years [28]. Similarly, research conducted at Majmaah University in Saudi Arabia reported higher EI levels among older students and those in advanced years of study [1]. According to one study, EI improves with age, supporting the notion that EI is a skill that can be developed rather than an innate, unchangeable trait [29]. Opposing research among Jordanian nursing and midwifery students found that increased age was associated with decreased EI scores, suggesting a complex relationship between age and emotional intelligence [30].

Some studies suggest that female students exhibit higher EI levels, while others find no significant gender differences. For instance, research from Majmaah University found substantial differences in EI levels according to gender [1]. Conversely, other studies have reported no significant correlation between gender and EI [29], which supports the current study findings. Marital status has also been associated with variations in EI. The study at Majmaah University found significant differences in EI levels according to marital status, with married students exhibiting higher EI scores [1]. Students reporting higher grade point averages (GPAs) tend to have elevated EI scores. The study at Majmaah University reported significant differences in EI levels according to GPA [1].

Furthermore, the results showed that reflective thinking was positively influenced by emotionality, self-control, sociability, and well-being. A study on nursing students highlighted that those with higher emotional intelligence, particularly in emotionality, demonstrated enhanced critical thinking skills integral to reflective practice. Research has shown that nursing students with higher self-control exhibit better clinical decision-making abilities, suggesting a link between self-control and reflective thinking [31]. Another study found a relationship between the total emotional intelligence score and clinical competence, which might enhance the students’ well-being [32].

### Limitations

This study has several limitations, even though it offers insightful information about the connection between nursing students’ reflective thinking and EI. First, the cross-sectional design makes it more difficult to prove a causal link between reflective thinking and emotional intelligence. Longitudinal research is required to investigate how these characteristics change over time. Second, when self-reported data is used, participants may overestimate or underestimate their abilities, which could lead to response bias. Thirdly, the study was limited in its applicability to other nursing student populations in diverse educational or cultural contexts because it was limited to a single Egyptian university. Furthermore, this study did not examine other factors affecting EI and reflective thinking, such as cultural background, socioeconomic level, or past life events. Lastly, although the tools used in the study have demonstrated reliability and validity, they may not fully capture the complexity of EI and reflective thinking in diverse nursing contexts.

## Conclusion and recommendations

Nursing students demonstrate high levels of EI and reflective thinking, with a significant positive relationship between the two. Research indicates that emotional intelligence is a strong predictor of reflective thinking. Therefore, incorporating EI training for nursing students is essential, as it enhances their reflective thinking capabilities and equips them for diverse clinical environments. Embedding concepts and domains of emotional intelligence into nursing education is crucial to fostering reflective thinking and ready students for effective patient care across various clinical scenarios. Ongoing training and education on emotional intelligence and its influence on reflective thinking should be provided to nursing students. This could include workshops, seminars, or online training to enhance self-awareness, empathy, and interpersonal skills, vital components of emotional intelligence.

Additionally, developing evidence-based strategies, protocols, or interventions will aid nursing students in cultivating their emotional intelligence and reflective thinking skills in clinical practice. Future research will utilize various sampling methodologies across multiple universities and implement longitudinal study frameworks. This broader approach will enhance the findings’ applicability and ensure that nursing practice recommendations are relevant in diverse settings.

### Implications for nursing and health policy

Nursing curricula should incorporate emotional intelligence (EI) training as a foundational component, as EI is linked to reflective thinking and decision-making. Integrating EI modules will help nursing students develop emotional competencies to manage clinical stress. Practice Implication: Educational institutions should guide students in emotional self-regulation, empathy, and interpersonal skills to enhance reflective thinking and clinical decision-making.

Healthcare systems should advocate EI-focused workshops for nurses, improving stress management and decision-making. Practice Implication: Emotionally equipped nurses experience less burnout, improving retention, morale, and patient care. Hospitals should embed reflective practices into professional development. Reflection enhances clinical decision-making and patient care. EI should be a key competency in nursing leadership selection. Higher EI fosters effective leadership, teamwork, and clinical outcomes.

## Data Availability

The datasets used or analyzed in this study are the corresponding authors upon reasonable request.
